# Discrimination between lectins with similar specificities by ratiometric profiling of binding to glycosylated surfaces; a chemical ‘tongue’ approach†
†Electronic supplementary information (ESI) is available: This includes protein preparation, surface functionalisation and LDA analysis. See DOI: 10.1039/c5ra08857g



**DOI:** 10.1039/c5ra08857g

**Published:** 2015-06-18

**Authors:** L. Otten, M. I. Gibson

**Affiliations:** a Department of Chemistry , University of Warwick , Gibbet Hill Road , Coventry , CV4 7AL UK . Email: m.i.gibson@warwick.ac.uk

## Abstract

Carbohydrate–lectin interactions dictate a range of signalling and recognition processes in biological systems. The exploitation of these, particularly for diagnostic applications, is complicated by the inherent promiscuity of lectins along with their low affinity for individual glycans which themselves are challenging to access (bio)synthetically. Inspired by how a ‘tongue’ can discriminate between hundreds of flavours using a minimal set of multiplexed sensors and a training algorithm, here individual lectins are ‘profiled’ based on their unique binding profile (barcode) to a range of monosaccharides. By comparing the relative binding of a panel of 5 lectins to 3 monosaccharide-coated surfaces, it was possible to generate a training algorithm that enables correct identification of lectins, even those with similar glycan preferences. This is demonstrated to be useful for discrimination between the cholera and ricin toxin lectins showing the potential of this minimalist approach for exploiting glycan complexity.

## Abstract 2

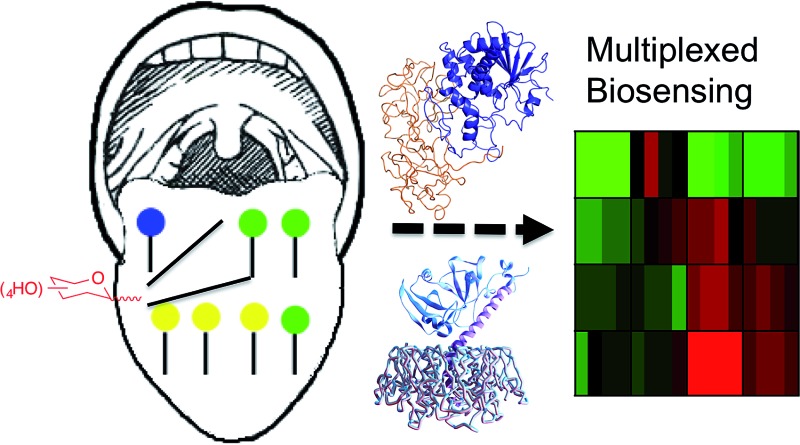
Glycan–lectin interactions drive infectious processes, but are characterized by relatively low specificity, especially for monosaccharides. Here we use multiplexed biosensing to discriminate between lectins (including cholera toxin).

## Introduction

Protein carbohydrate interactions are essential for many biological processes including cell–cell communication, fertilisation and innate immunity.^1^ They are also readily exploited by pathogens during adhesion steps. These adhesion steps are mediated by carbohydrate binding proteins known as lectins. In nature, multivalent presentation of glycans at cell surfaces increases the affinity towards its binding partner which has been widely exploited to create synthetic glycomimetics, such as glyco-polymers^2^ and particles.^3–5^ This strategy however does not necessarily maintain or improve the selectivity complicating the design and application of multivalent glycoconjugates.^2,5–8^

Lectin interactions are mediated by the carbohydrate itself but also the linker between the carbohydrate, the cell surface and precise 3D presentation of carbohydrates on the cell surface.^6,9^ Many lectins show highly specific binding to oligosaccharides but show much more promiscuous binding characteristics on a mono- and di-saccharide level. For example, peanut agglutinin (PNA) is generally described as being β-galactose specific but microarray analysis shows that it will readily bind all monosaccharides with very little difference between them.^10^ The same is also true for cholera toxin, this toxin is highly specific to the GM-1 ganglioside in the body and thus is described as being galactose specific but this lectin will indiscriminately bind all monosaccharides to one degree or another.^10^

This wide variety of roles played by glycans in the body's innate processes and their prevalence in nature means the interference or detection of these interactions could have an impact in combatting infectious diseases.^11^ For example, FimH is a lectin involved in the binding of uropathogenic *Escherichia coli* to mannose rich residues and is a crucial virulence factor. Cholera is caused by cell internalisation of an AB_5_ toxin, mediated by the 5 lectin subunits of the toxin initiating binding to GM-1 on epithelial cells in the small intestine. Ricin is a toxic protein extracted from *Ricinus communis* seeds, it consists of one subunit responsible for cleaving an adenine residue from the 28S ribosomal RNA (thus rendering the cell incapable of protein synthesis) and one subunit responsible for binding to galactose rich residues.^12^ Differences in glycosylation of cells have also been implicated in tumour cells and determining metastatic potential of cancers^13,14^ and the ABO blood system is also determined by different antigenic oligosaccharides.^11,15^ Serological blood groups have been implicated in individual susceptibility to many diseases and the severity of others including small pox, cholera and malaria.^15–17^ As such rapid detection of lectins can aid in the early identification and prevention of diseases and also in the design of therapeutics. This broad window of binding partners means that the design of a sensor for a lectin based on glycans alone is immensely challenging.

Whilst proteomic and antibody based techniques can be used for identification of lectins these are not always suitable for robust, point of care applications, and require infrastructure for preparation, storage, distribution and deployment of the sensor. Such a challenge is indeed not unique to glycobiology, and the detection of cell phenotypes, which often have dynamic surface ligand displays which change with their environment. To address this nanoparticles multiplexed biosensing has attracted much interest especially for diagnostics.^18^ Rotello *et al.* have developed the use of differentially functionalised gold nanoparticles for multiplexed diagnostics. For example, 52 different mixtures of seven different proteins could be identified using just six distinct nanoparticles.^19^ Gold particles coated with 3 different thiols enabled cancerous and healthy cells to be discriminated without the requirement for any specific binding epitopes.^20^ Detection of pathogenic bacteria using a related system in under 5 minutes has also been demonstrated^21^ as have MRI based detection of cancerous cells with differential lectin expression levels.^22^ Jayawardena *et al.* have described the use of glycosylated gold nanoparticles and their characteristic shift in SPR frequencies upon protein binding to characterise lectins based on their response to a panel of sugars.^23^ In this case, lectins with very different glycan specificities were used (*e.g.* concanavalin A/soybean agglutinin) and discrimination was also possible without the need for multiplexing and just using individual glycans making it a less challenging analysis.

The goal of the present research was to evaluate the use of simple and synthetically accessible mono-saccharides as multiplexed sensors to enable discrimination between different lectins which have similar binding specificities. Such a system would have widespread application especially for low-cost selective detection/monitoring of toxins.

## Results and discussion

**Fig. 1:**
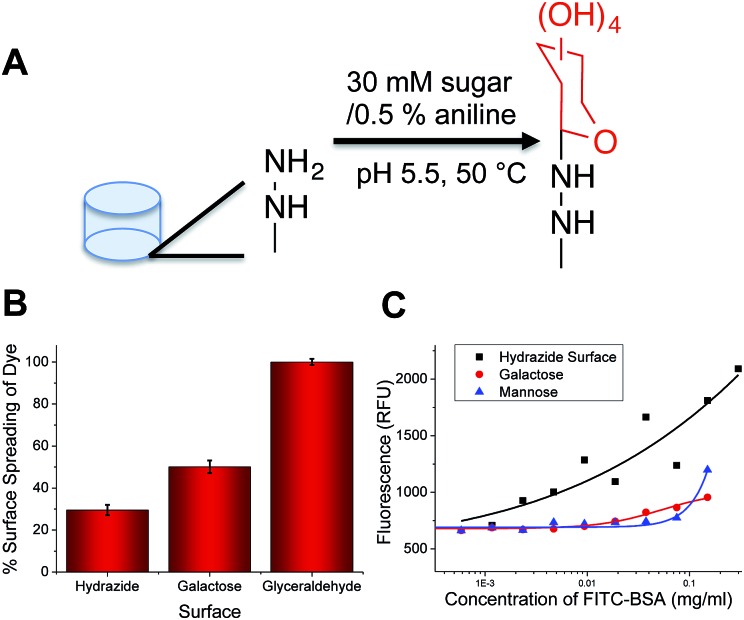
Fabrication of glycosylated 96-well plates. (A) Hydrazide–carbohydrate coupling; (B) dye spreading assay showing relative hydrophilicity of surfaces; (C) non-specific binding of fluorescently-labelled bovine serum albumin onto different surfaces following 30 minutes incubation and washing.

The key aim of this work was to probe the differential response of lectins to simple carbohydrates (monosaccharides), so it was essential to employ accessible (/facile) coupling chemistry. 96-multiwell plates with hydrazide functionality were used to couple a range of monosaccharides, and mixtures of different monosaccharides using an aniline catalyst at 50 °C to give glycosylated surfaces (Fig. 1A). This coupling mechanism is known to result in attachment of the monosaccharides predominantly in their ring closed (pyranose) β-anomeric form.^24^ It should be noted that the presence of some acyclic species does not affect our later analysis using a training algorithm (*vide infra*). It was not possible to interrogate the functionalised polypropylene surface of the microwell plate using traditional surface analysis techniques (such as elipsometry). As an alternative to contact angle, droplet spread was measured. Hydrophobic surfaces, when viewed from above should give reduced surface coverage at equal volume, compared to a hydrophilic surface with a low contact angle. The native, and glycosylated surfaces were therefore interrogated by addition of a drop of ultra-pure water with resorufin (a dye) added to enable visualisation of the droplet spread, and subsequent image analysis. The native surfaces resulted in only 30% of the surface being covered by the drop, but the galactose functional surface resulted in droplet spreading over 50% of the surface (Fig. 1B). As a positive control, glyceraldehyde was added to generate very hydrophilic surface coating, and this resulted in spreading over ∼90% of the surface, confirming this (unconventional) analytical approach. Glycan-modified surfaces should also present an uncharged, non-fouling surface, compared to the native hydrazide/polypropylene surface. Therefore, non-specific fouling (adsorption) was tested using FITC-labelled bovine serum albumin. Compared to the native surface, the glycosylated surfaces showed significantly less binding than the native surface, with no significant absorption observed at concentrations below 0.2 mg mL^–1^ again confirming the surface modification (Fig. 1C).

**Fig. 2:**
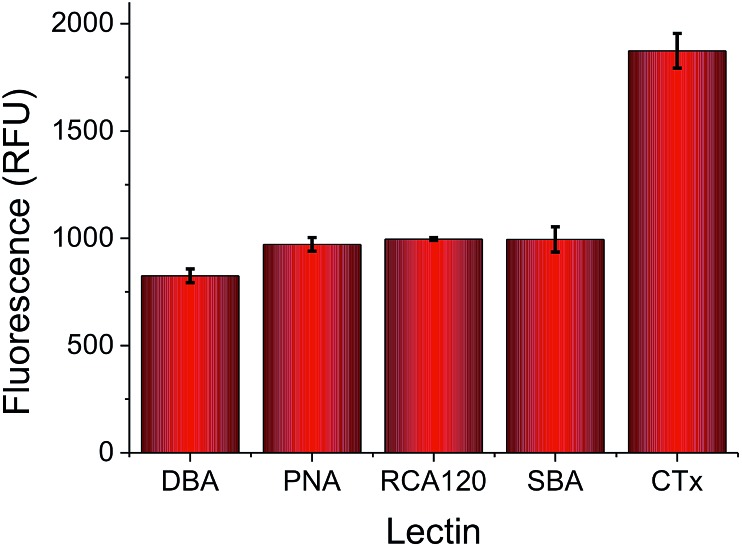
Relative binding of a panel of 5 lectins to a galactose-functional surface as judged by fluorescence intensity. All lectins applied at 0.01 mg mL^–1^, with FITC labels.

To highlight the challenges faced in identification and profiling of lectins with similar binding specificities, a panel of 5, fluorescently labelled, galactose (or GalNAc) binding lectins were selected, exposed to a galactose microwell plate, washed and total fluorescence measured. Fig. 2 shows the results of this, indicating that at any given concentration the total response recorded is not unique to any given lectin. Cholera toxin B subunit (CTx) gives higher binding than the others, but the absolute fluorescence intensity is obviously dependent on the concentration applied, which is not ideal for any realistic biosensory format as it requires significant prior knowledge of the solution being probed.

**Fig. 3:**
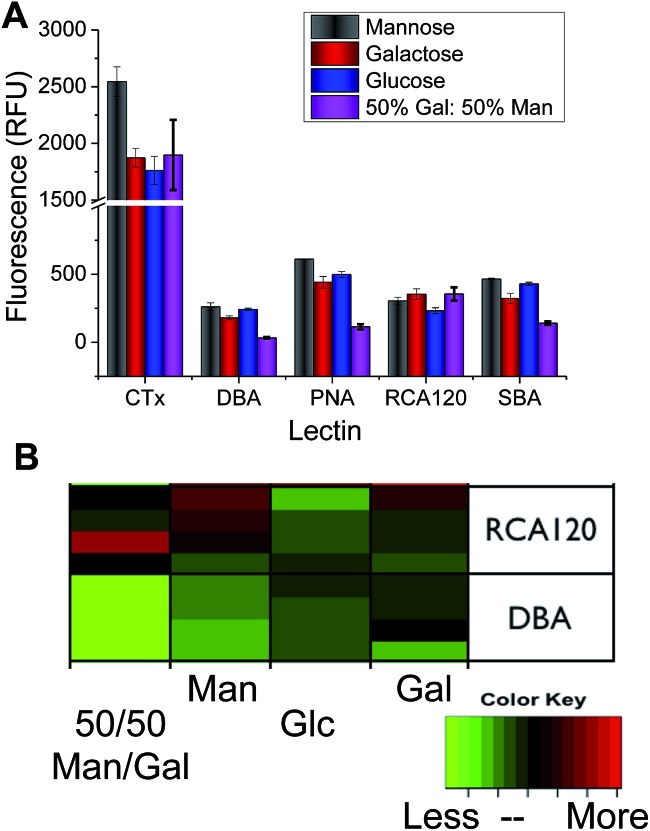
(A) Relative lectin binding to glycosylated surfaces determined by fluorescence; (B) heatmap, demonstrating that each protein has a ‘barcode’ of responses to each glycan, each lectin displayed contains at least 4 independent replicates.

Considering the low information content of these single sugar assays, we proceeded to extract information for a series of Gal-binding lectins from the CFG database (consortium for functional glycomics) to a range of small mono/disaccharides (figure of this analysis included in ESI†). The CFG data revealed that any single glycan cannot predict the identity of the lectins (*i.e.* a single peak is not present) due to their inherent promiscuity. However, if many different glycans are included, there is a unique pattern of binding of each lectin to the carbohydrates (a ‘barcode’). Guided by this data, we rationalised that if we could identify the ‘minimum basis set’ of glycans that can provide a unique barcode for each lectin, it would be possible to distinguish between these, enabling protein identification without proteomics or associated methods. Using the hydrazide coupling chemistry described above, we generated 4 differently glycosylated surfaces; Gal, Man, Glc and a 1 : 1 mixture of Gal : Man (the latter was added as in our hands this improves the resolution of our subsequent analysis. Variable density glycan mixtures are known to give non-linear responses^7^). Pleasingly, these relatively low-affinity monosaccharides produced very unique binding profiles for each lectin, as shown in Fig. 3. For example, *Ricinus communis* Agglutinin (RCA_120_) had significantly higher binding to galactose, and the Gal/Man mixtures, than compared to Glc binding. Conversely, Soybean Agglutinin (SBA) had significantly depressed binding to the mixed surface. A summary of the relative binding of the lectins can be shown in a heat map to give a ‘bar-code’ which is unique to each protein.

Analysis of the individual binding of one lectin to a sugar does not give much information, but when combined together, this differential response provides sufficient information to enable a linear discriminant analysis. Linear discriminant analysis is a training algorithm that inputs a matrix of data and produces a model in which all of the categories in the initial training matrix are grouped into distinct categories based on their linear discriminant factors (which are a linear combination of the initial inputs-in this case the surfaces used). Due to the high degree of separation between categories within the model produced it allows for greater confidence in the identification of lectins responsible for binding in unknown samples when compared to the raw data alone.

**Fig. 4:**
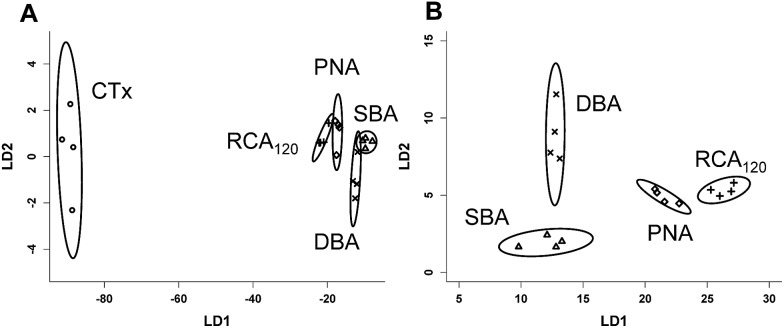
Linear discriminant analysis of lectin binding to the 4 different glycosylated surfaces. (A) Lectins with CTx, and (B) lectins without CTx.

Fig. 4A shows the results of a linear discriminant analysis of these lectins to the four glycosylated surfaces, revealing highly resolved groupings for each lectin. The circles around each are indicative of a 95% confidence boundary. Fig. 4B shows the LD analysis for the lectins without CTx, as when this is included the other four lectins appear more tightly bunched (but are still perfectly resolved) due the generally increased binding of CTx to all surfaces employed here. This simple, but powerful, multiplexed method enables separation and identification of lectins with similar binding profiles, but without the need for complex carbohydrates, in much the same way as a tongue has evolved to identify complex tastes based on only 5 different inputs. To test the predictive power of this, blind analysis of unknown lectin samples was also conducted, revealing 100% predictive accuracy from this training matrix.

As a final test of this sensing approach, the differentiation between two different gal-binding, pathogenic, lectins was investigated. CTx is the toxin secreted by the bacteria *Vibrio cholera*, which causes cholera and is a huge problem in developing countries and disaster zones. RCA_120_ is a surrogate for ricin, which can be weaponised as a biological warfare agent. A training algorithm was again employed, but this time the RCA_120_/CTx solutions were applied as mixtures of the two lectins, rather than as pure protein solutions. This provides a far more challenge test, which is closer to a real world sensing application. When CTx was present at > 50% (by mass) the LD model correctly indicated its presence, and when the RCA_120_ concentration was above 50%, this was correctly scored (see ESI† for full details and LDA graphs).

## Conclusions

Here we have reported the new concept of a ‘chemical tongue’ for multiplexed biosensing, and discrimination between carbohydrate-binding proteins (lectins). We show that using only simple monosaccharides, which have very low intrinsic affinity and specificity, it is possible to discriminate between a panel of lectins with extremely similar binding preferences. The power of this method lies in the scalability, enabling many more (oligo)saccharides to be employed, and the use of the large glycan databases (which are freely available) to guide the design of each system. Using this approach we demonstrated that the chemical tongue can even distinguish the presence of cholera or ricin, in complex mixtures of the two lectins. Current and future work is focused on establishing the limits and scope of this method, and translating it into realistic sensory surfaces/components which would remove the need for labelled proteins.

## Experimental

Full experimental detail including microplate functionalization LDA analysis is provided in the ESI.†
