# Supplementary material for: Discrimination between lectins with similar specificities by ratiometric profiling of binding to glycosylated surfaces; a chemical ‘tongue’ approach
Source: RSC Adv. 2015 Jun 18;5(66):53172–9. doi: 10.1039/c5ra08857g (PMC4786966; doi:10.1039/c5ra08857g)
Supplement: Supplementary file 1 [file RA-005-C5RA08857G-s001.pdf]

Electronic Supplementary Material (ESI) for RSC Advances.
This journal is © The Royal Society of Chemistry 2015

Supporting Information for

Discrimination between Lectins with Similar Specificities by Ratiometric Profiling of Binding to Glycosylated Surfaces; A Chemical 'Tongue' Approach

L. Otten and M. I. Gibson [a]

[a] Department of Chemistry, University of Warwick, Gibbet Hill Road, Coventry, CV4 7AL UK.

*Corresponding author email; m.i.gibson@warwick.ac.uk

Materials and Methods Section

Materials. All chemicals were used as supplied unless otherwise stated. Corning® 96 well clear flat bottom polystyrene Carbo-BIND™ microplates, aniline (99.5 % ACS reagent), D-(+)mannose, α-D-glucose, D-(+)galactose, bovine serum albumin, fluorescein isothiocyanate isomer 1 (≥ 90% HPLC), DMSO, PD10 desalting columns, phosphate buffered saline tablets and FITC conjugated Cholera Toxin B subunit were purchased from Sigma-Aldrich. 100 mM acetate buffer with 1 mM aniline (pH 5.5) was prepared in 200 mL of milliQ water (with a resistance >19 mOhms). 10 mmol HEPES buffer containing 0.05 M NaCl, 0.1 mM $CaCl_2$ and 0.01 mM $MnCl_2$ (pH 7.5, HEPES) was prepared in 200 mL of milliQ water (with a resistance >19 mOhms). FITC labelled *Dolichos biflorus* agglutinin, Soybean agglutinin, *Ricinus communis* Agglutinin I and peanut agglutinin were purchased from Vector Labs.

Preparation of glycosylated surfaces. 30 mM monosaccharide solutions were prepared in 100 mM acetate buffer with 1 mM aniline. 100 μL of monosaccharide solution was then added to every well of a hydrazide functionalised 96 well microtitre plate before incubation at 50 °C for 24 hours. After incubation wells were extensively washed three times with MilliQ water before being allowed to dry. Plates were functionalised with either galactose, glucose, mannose or a 1:1 solution of galactose and mannose and were either used immediately or stored at -20 °C .

Surface functionalization analysis. 10 μL droplet of 1 $mg.mL^{-1}$ resorufin in water is added to the well surface. A picture is then taken of the droplet inside the well using a

smartphone. The image is imported into ImageJ (version 1.46a) and the surface area of the well covered by the droplet is determined by drawing regions of interest around the well and the droplet and converting the area of the droplet region of interest into a percentage relative to the area of the well region of interest. This procedure was completed for a hydrazide functionalised surface and the same surface after additional functionalisation with glyceraldehyde and galactose.

Lectin binding assays. Various fluorescently labelled lectins (DBA, CTx, PNA, RCA120 and SBA) were dissolved to a concentration of 0.01 $mg.mL^{-1}$ in 10 mM HEPES with 0.15 M NaCl, 0.1 mM $CaCl_2$ and 0.01 mM $MnCl_2$ (pH 7.5). 100 μL of the lectins were then added to each well of a microtitre plate before incubation in the dark at 37 °C for 30 minutes. After incubation, each well was rigorously washed 3 times with water before fluorescence readings were taken. Fluorescence readings were taken using a BioTek Synergy HT multi-detection microplate reader and Gen5 software with excitation and emission wavelengths of 485 and 528 nm respectively.

Blind sample assays. Blind samples were prepared in the same method as the lectin binding assays. Briefly, 100 μL of each blind sample (with a lectin concentration of 0.01 mg/mL) was added to each well and after incubation in the dark at 37 °C for 30 minutes wells were washed with water before fluorescence readings were taken. Fluorescence readings were taken using a BioTek Synergy HT multi-detection microplate reader and Gen5 software with excitation and emission wavelengths of 485 and 528 nm respectively.

Linear discriminant analysis. Every lectin was added to each surface as described in the lectin binding assay section. This was repeated 4 times to generate a training data matrix of 4 surfaces x 5 lectin x 4 replicates, which was then subjected to a classical linear discriminant analysis (LDA)[1] in the open source statistical package R (version 2.14.1).[2] The model produced in this analysis was used to predict the nature of the random samples using the predict function in R.[1]

Heatmap production. A heatmap of fluorescence readings was produced by displaying every individual fluorescence reading as a colour based on a scale using the heatmap() function in the open source statistical package R (version 2.14.1). [2,3] The Heatmap was produced both with CTx and without for ease of viewing in the main text. CTx showed far higher binding to the surfaces (which may also be due to extent of labelling differences) meaning that the other 4 lectins give heat map results in the red area of the spectrum, which are mathematically distinct but harder to visualise. It is included here in the supporting information for completeness, but for clarity, the smaller version is in the main text.

Fluorescently labelled bovine serum albumin. A stock solution of 1 mg/ml BSA in PBS was prepared and 30ul of 10 mg/ml of FITC in DMSO was added to every ml of BSA solution. This was then incubated at room temperature in the dark for 1 hour whilst stirring continuously. Before use, the solution was washed through a PD10 desalting column in order to remove unlabelled protein and unbound dye.

Non-fouling surface analysis. 100 μL of FITC-BSA was added as a serial dilution (from 1 mg.mL^{-1} in PBS) to the wells of both unfunctionalised hydrazide 96-well

plates and those functionalised with galactose or glucose. Fluorescence measurements were then taken using a BioTek Synergy HT multi-detection microplate reader with Gen5 software using excitation and emission wavelengths of 485 and 528 nm respectively (and a sensitivity of 75 nm).

Linear discriminant analysis plots. LDA plots were drawn in R using the plot function. Each data point represents the fluorescence readings for a single lectin transformed into linear discriminants using the LDA model. Ellipses were added using the ellipse function and represent the mean linear discriminant values for each lectin category +/- the standard deviation of the linear discriminants.

Additional Data

Consortium for Functional Glycomics binding profiles. Binding profiles for a series of Gal binding lectins interacting with a range of mono- and di-saccharides was extracted from the CFG database (all lectins were at 10 µg/ml). As can be seen by Figure S1, it would be challenging to tell each lectin based on binding to a specific glycan but upon considering a large sample of glycans a unique 'barcode' can be produced for each lectin.

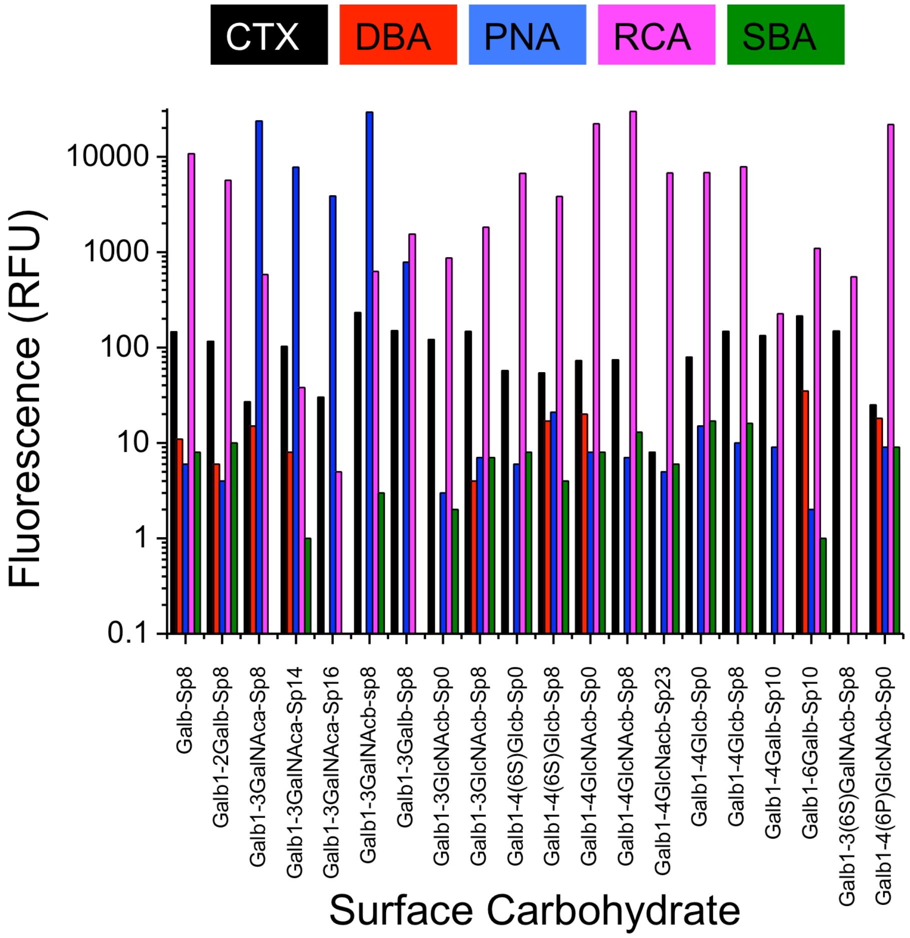

Figure S1. Glycan microarray analysis showing relative affinity a range of sugars to different Gal-specific lectins. Data is expressed as total fluorescence.

Heatmap of lectin binding. Heatmap showing relative binding profiles of 4 lectins to several surfaces is shown in Figure S2. A unique profile can clearly be identified for each lectin.

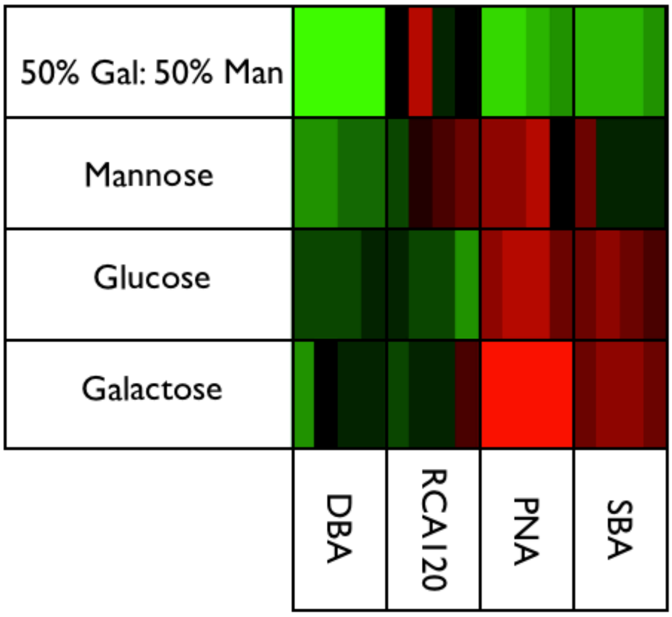

Figure S2. Heatmap, demonstrating that each protein has a 'barcode' of responses to each glycan the colours indicate the fluorescence reading achieved for each individual sample of that lectin added to the surface.

Linear discriminant analysis of unknown samples Table 1 shows the results of identification of blind samples using the model produced using linear discriminant analysis. 100 % predictive accuracy of blind lectin samples was found using this model.

Table 1. LDA analysis of blind lectin samples.

Blind Sample	Prediction	Actual
U1	RCA120	RCA120
U2	PNA	PNA
U3	SBA	SBA
U4	DBA	DBA
U5	CTx	CTx

Linear discriminant analysis of mixed samples Figure S3 shows the binding intensity (total fluorescence) of the lectin mixtures in a range of ratios. When CTx was present at > 50 % (by mass) the LD model correctly identified its presence, and when the RCA120 concentration was above 50 %, this was correctly scored (Table 2). This simplified experiment demonstrates the potential of our method, which has the potential to be applied to other sensory surfaces, and other lectins by simply exchanging the carbohydrates which are employed. Future work will focus on integrating this to real sensors and exploring the scope of the discriminatory power of this method, especially towards water-borne pathogens.

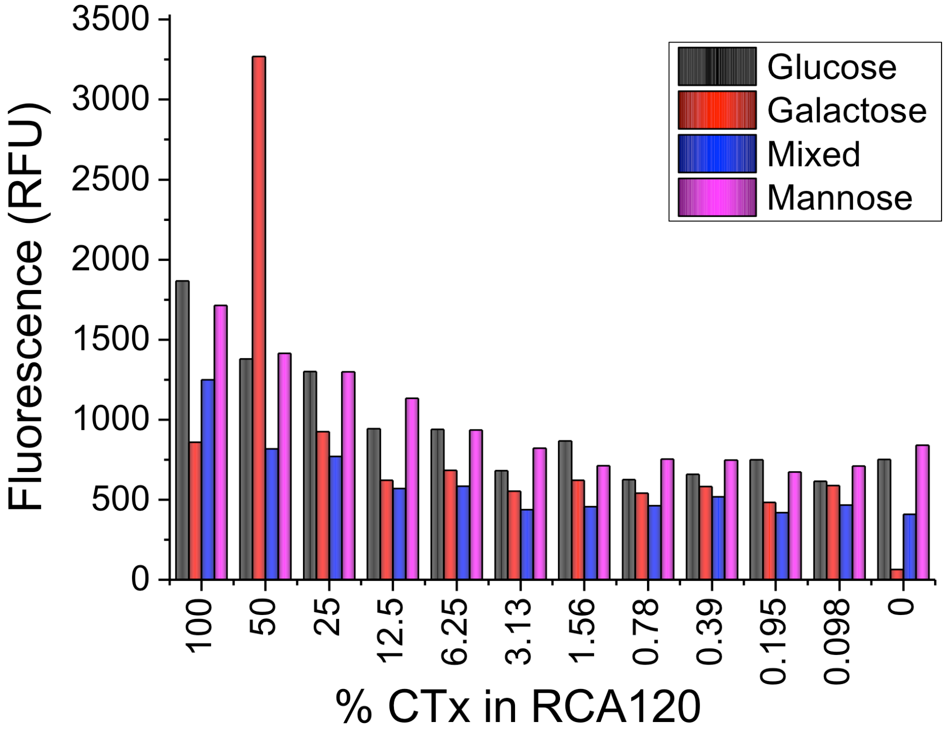

Figure S3. Binding profile of a range of mixtures of CTx and RCA120 and results of blind testing of mixtures based on the LD training algorithm.

Table 2. LDA analysis of CTx and RCA_{120} mixtures.

	**Lectin Prediction Result**
**Percentage of CTx in RCA_{120}**	**CTx**
100	✓
50	✓
25	✗
12.5	✗
6.25	✗
3.125	✗
1.563	✗
0.781	✗
0.391	✗
0.195	✗
0.098	✗
0	✗

References

1. Jonathan Chang (2011). R package version 1.3.1.http://CRAN.R-project.org/package=lda
2. R Development Core Team (2011). R Foundation for Statistical Computing, Vienna, Austria. ISBN 3-900051-07-0, http://www.R-project.org/
3. Murdoch, D.J. and Chow, E.D. (1996). A graphical display of large correlation matrices. The American Statistician 50, 178-180.
